# Optic neuropathies post-Covid 19 - review

**DOI:** 10.22336/rjo.2022.54

**Published:** 2022

**Authors:** Sorina-Elena Abdul-Salam (State), Veronica Sfredel, Carmen Luminița Mocanu, Carmen Valeria Albu, Andrei-Theodor Bălășoiu

**Affiliations:** * Department of Physiology, University of Medicine and Pharmacy of Craiova, Romania; ** Department of Ophthalmology, University of Medicine and Pharmacy of Craiova, Romania; *** Department of Neurology, University of Medicine and Pharmacy of Craiova, Romania

**Keywords:** optic neuropathy, Covid 19, neuro-ophthalmological manifestations

## Abstract

The Corona virus infection started at the end of 2019 in Wuhan - China and spread rapidly throughout the world, generating the Covid 19 pandemic. The manifestations of the Covid disease were extremely varied, from a simple flu, with fever, cough, weakness, headache, joint pain, up to severe pneumonia, with severe acute respiratory syndrome (SARS-Cov2) and even death. The symptomatology of the disease, the evolution and the complications that appeared varied, depending on the associated pathology - diabetes mellitus (DM), hypertension (HT), the age and the immune status of the patient.

**Aim:** The ocular manifestations related to Covid 19 were mostly represented by conjunctivitis, but the neurotropic character of Corona virus could justify the appearance of certain neuro-ophthalmological manifestations, such as: optic neuritis (ON), cranial nerve palsies, visual field (VF) anomalies. The aim of this paper was to research the cases of optic neuropathy post-Covid 19, published in the specialty literature between 2020 and 2022. The following were evaluated: risk factors, distribution by age group and gender, evolution and complications, as well as the clinical forms of optic neuropathies.

**Materials and methods:** We used Google Scholar and PubMed databases to find articles on optic neuropathies related to the Covid-19 infection. We followed the articles published during the pandemic and selected 21 cases, belonging to 17 authors, irrespective of their origin and the language in which they were written.

**Results:** 21 patients affected by ON in the Covid-19 disease, 11 women and 10 men, were mentioned. The optic neuropathies described by the authors were: retrobulbar optic neuropathy, only one associated with myelin oligodendrocyte glycoprotein (MOG), papillitis, neuroretinitis, anterior ischemic optic neuropathy (AION), out of which one arteritic anterior ischemic optic neuropathy (AAION) and the others non-arteritic anterior ischemic optic neuropathy (NAAION), one being related to pronation in an oro-tracheal intubated (OTI) patient with acute respiratory distress syndrome (ARDS).

**Discussions:** The neuro-ophthalmological complications associated with Covid 19 disease can be severe, so the patients should be monitored continuously. Many investigations (serological, immunological and imaging exams) are necessary to exclude other etiologies of ON.

**Conclusions:** A complete ophthalmological exam is mandatory for each patient diagnosed with Covid 19 disease, even if they have ocular manifestations or not.

**Abbreviations:** SARS-Cov2 = severe acute respiratory syndrome; DM = Diabetes mellitus; HT = Hypertension; ON = Optic neuritis; VF = Visual field ; NS = Nervous system; CRP = C-reactive Protein; CL = cytokines; IL = interleukins; TNFɑ = tumor necrosis factor; CNS = central nervous system; ACE = angiotensin-converting enzyme; CRVO = central retinal vein occlusion; MOG = myelin oligodendrocyte glycoprotein; MOG-AD = myelin oligodendrocyte glycoprotein antibody disease; BBB = blood-brain barrier; ARDS = acute respiratory distress syndrome; IOP = intraocular pressure; CVP = central venous pressure; MSOF = multiple systems organ failure; AAION = arteritic anterior ischemic optic neuropathy; NAION = non-arteritic anterior ischemic optic neuropathy; AION = anterior ischemic optic neuropathy; OCT = optical coherence tomography; CT = computer tomography; AFG = angiofluorography; MRI = magnetic resonance imaging; ESR = erythrocyte sedimentation rate; RF = rheumatoid factor; ANA = antinuclear antibodies; ANCA = antineutrophil cytoplasmic antibodies; AQP4 = anti aquaporin 4; NMO = neuromyelitis optica; CSF = cerebrospinal fluid; OTI = oro-tracheal intubated; VA = visual acuity; ONTT = optic neuritis treatment trial; RNFL = retinal nerve fiber layer; ICU = intensive care unit; LE = left eye; RE = right eye; MS = multiple sclerosis; ICH = intracranial hypertension; BCVA = best correction visual acuity; LP = light perception; APD = afferent pupillary defect; BM = biomicroscopy; PDN = prednisone; MTX = methotrexate; MTPN = methylprednisolone; NSAID = non-steroidal anti-inflammatory drugs; CGL = cells ganglion layer; VEP = visual evoked potential; CF = counting fingers

## Introduction

Optic neuropathies represent a vast chapter of ophthalmology, with multiple etiopathogenic mechanisms, varied, sometimes unpredictable evolutions and often irreversible complications. The neurotropic and neuroinvasive character of the Corona virus was highlighted throughout the COVID-19 pandemic by the installation of various forms of optic neuropathies in infected patients. Sars-Cov-2 infection can affect the nervous system (NS) through different mechanisms: either through a direct, neurotoxic action of the Corona virus, or through an immune mechanism of breaking the blood-brain barrier (BBB), or through a disruption of the coagulation system with hypercoagulability and the formation of blood clots [**1**].

## Materials and methods

The article proposed a synthesis of several cases of post-Covid optic neuropathy, which were published in the specialty literature between 2020 and 2022. 17 publications analyzing 21 cases of ON were encountered, using the PubMed and Google Scholar search engines, by entering the keywords: optic neuropathy post Covid, neuro-ophthalmological manifestations, ON. 

**Table 1** presents the data related to the authors of the articles, as well as all the details regarding the age and gender of the patient, the form of the Covid disease, the interval between the confirmation of the Covid-19 disease by a positive nasopharyngeal PCR test and the appearance of ocular symptoms, ocular manifestations, the ophthalmological examination and the evolution of optic neuropathies after treatment. 

**Table 1:** Details of all cases of NO

The authors of the study	Age/ sex	Duration between positive PCR Covid test and ocular symptoms (weeks)	Covid 19 form and comorbidities	Ocular signs and symptoms	Diagnosis	Evolution and treatment
Szydełko-Paśko U et al. [**2**]	69/ F	2,5 weeks	Mild form; without hospitalization, just fever, cough, fatigue. Comorbidities: HT, well-controlled type II DM	BCVA RE = 0.9; BCVA LE = nasal and superior LP, without APD in RE; LE: pupil is reactive to direct light, slowly reactive to indirect light; IOP in RE = 10 mmHg, IOP in LE = 09 mmHg. BM: Cortical cataract RE; Fundus RE: cotton wool spots in the upper arterio-venous sector. Fundus LE: blurring of the optic margins with flame hemorrhages; AFG: delayed choroidal filling with watershed areas	LE: AAION in the course of giant cell arteritis after Covid 19	Enoxaparin 0.4 ml/ day for 10 days then PDN 80 mg/ day for 4 days. At discharge: MTX 15 mg/ week and PDN with tapering doses. At 3 months: BCVA RE = 0.8, BCVA LE = LP; OCT: macular traction, RE: normal optic disc, LE: thinning RNFL with optic atrophy.
Kitson D et al. [**3**]	21/ F	1 week	Mild form; ocular onset with accidental discovery of PCR +	BCVA RE = 20/ 20, BCVA LE = HM. IOP-RE = 21 mmHg, IOP-LE = 23 mmHg; Fundus RE: mild temporal burring of papilla with nasal sparing; Fundus LE: papilledema	LE: Unilateral ON associated with Sars-Cov-2 infection	MTPN, remdesivir and oral steroid at discharge. VA returned to normal after 5 days of treatment; after one month: normal VF and eye fundus.
Sawalha K et al. [**4**]	44/ M	2 weeks	Mild form; shortness of breath and cough, ambulatory symptomatic treatment	Bilateral eye pain, decrease of VA in both eyes 7 days before presentation to the ophthalmologist. BCVA RE = 20/ 200 with RAPD and global loss of VF; BCVA LE = 20/ 30 and superior arciform deficit of VF	Acute bilateral ON induced by Covid19	MTPN 1 g/ day for 5 days, then PDN 1 mg/ kg/ day for 11 days and another 4 days with tapering doses. After 48 hours: improved VA and pain relieved.
Saray Rodriguez M [**5**]	55/ F	2 weeks	Mild form; without respiratory symptoms	Exacerbated headache and eye pain when moving the eyeball in LE for approximately 12 days, despite NSAID treatment; gradual decrease in VA and chromatic impairment. BCVA RE = 20/ 40; BCVA LE = 20/ 200 with RAPD; RE: normal OCT; normal VF; LE-VF: inferior centrocecal and centronasal scotoma.	LE: ON after infection with Sars Cov 2	Oxygen therapy 1 g/ day, MTPN iv for 5 days, then oral PDN with tapering doses. After one month: BCVA RE = 20/ 40, BCVA LE = 20/ 400 with RAPD. Fundus LE: optic disc pallor; VF - centrocecal scotoma; OCT: RNFL thinning in the temporal sector and CGL decrease; MRI of the orbit: mild increased thickness and signal in the left optic nerve. Normal brain MRI, normal inflammatory tests, negative PCR. CSF exam: IgG oligoclonal bands present in CSF but not in serum, anti-AQP4 antibodies negative in CSF and serum. After one month: LE: without eye pain; optic atrophy despite the treatment.
Žorić L et al. [**6**]	63/ M	4 weeks	Moderate form; fatigue, shortness of breath, fever 38 degrees, dry cough, bilateral bronchopneumonia with PCR “ – ” but IgM and IgG antibodies for Covid+; SpO2=92%; hospitalized for antibiotic and anticoagulant treatment (due to increased suspicion of Covid) Comorbidities: HT and DM (new case)	BCVA RE = 0.03: BCVA LE = 1; Fundus RE: small papilledema; predominantly right-sided headache, therefore, suspicion of AAION. OCT: partial swelling of the optic nerve head and CGL; RAPD.	RE: ON in a patient with MOG+ antibodies during the post-COVID-19	MTPN 1 g/ day iv for 5 days and PDN for 2 weeks after hospitalization. Day 5: BCVA RE = 0.3; improved VF; no headache. Brain MRI on day 7 with contrast: signs of microangiopathy and cortical reduction, normal orbits and optic nerves. After 3 weeks, the antigenic bands for IgG and IgM were negative, without neurological signs. BCVA RE = 20/ 25 (0.8), without papillary edema; BCVA LE: normal. After 2 weeks: MOG + (titer 1:40), cut off 1:10. After 3 months: BCVA RE = 20/ 20; normal VF; VEP: slight prolonged latency of the p100 wave; OCT: thinning of the RNFL and CGL. Titer of MOG antibodies was 1:20 and titer of IgM and IgG atc for Sars Cov 2 were 17.32, respectively 40.02.
Jossy A et al. [**7**]	16/ M	2 weeks	Mild form; isolation at home, without oxygen therapy or steroids.	Sudden loss of vision in LE 3 days before, accompanied by headache and pain when moving the left eyeball. BCVA RE = 20/ 20cc, BCVA LE = LP with RAPD; both eye fundus- normal, without edema or hyperemia	LE: Retrobulbar ON	ONTT scheme: MTPN iv for 3 days, oral PDN 1 mg/ kg for 3 days, with tapering doses over the next 3 days. Day 7: BCVA LE = 20/ 120; day 21: BCVA LE = 20/ 60; after 2 months: BCVA LE = 20/ 32.
Jossy A et al. [**7**]	35/ M	6 months before vision loss	Mild form; isolation at home, without oxygen therapy or steroids.	Sudden loss of vision in LE, accompanied by pain when moving the eyeball for 7 days. BCVA RE = 20/ 20, BCVA LE = 20/ 600 with mild APD in LE. Fundus LE- papillary and peripapillary edema, also confirmed on OCT; normal RE fundus.	LE: Papillitis	ONTT scheme: MTPN iv 1 g/ day for 3 days, followed by oral PDN with tapering doses. After 2 weeks: BCVA LE = 20/ 200 without any change in the following 2 months.
Jossy A et al. [**7**]	38/ M	6 weeks	Mild form; isolation at home, without oxygen therapy or steroids.	Sudden loss of vision in LE, pain when moving the eyeball for 5 days. The patient presented the same symptomatology a month ago, for which he underwent iv treatment with MTPN and oral PDN. The symptoms improved after a week; after 3 weeks VA decreased again. Normal fundus, eyes fundus.	LE Retrobulbar ON associated with MOG+ antibodies	ONTT scheme: MTPN iv 1 g/ day for 3 days, followed by oral PDN with tapering doses. Day 7: BCVA LE = 20/ 20; without any change in the following 2 months.
Sarwar S et al. [**8**]	47/ F	3 weeks	Moderate form; hospitalized 3 weeks before for Covid with fever, dyspnea, cough, myalgia, oxygen therapy due to level fluctuations. Comorbidities: unbalanced DM type II	Blurring of vision in LE in the last 18 hours; mild pain behind the eyeball, exacerbated by movement of the eyeball; well-oriented in time and place, anxiety, afebrile, blood pressure 110/ 80 mmHg, heart rate: 92 beats/ minute, SpO2 97%. BCVA LE = 60/ 200, BCVA RE = 20/ 20; RAPD to LE; fundus LE: mild edema.	LE: ON associated with MS after Sars-Cov-2 infection	Prednisolone 1 g/ day and symptomatic treatment; upon discharge, tapering doses of oral dexamethasone with improvement of VA and general symptoms.
Azab MA et al. [**9**]	32/ M	2 weeks	Severe form, hospitalized for 10 days in ICU for severe complications, PCR+	One week later, the patient presented a gradual decrease in vision with a central scotoma in the LE; headaches. BCVA RE = 20/ 30, BCVA LE = 20/ 200, LE: RAPD; IOP RE = 16 mmHg, IOP LE = 23 mmHg, BM both eyes: normal; color depth affection; LE fundus: mild disc swelling, without other retinal changes.	LE: ON post COVID19 infection	Without antiviral treatment; paracetamol 1 g/ day for 7 days, then MTPN iv 1 g/ day for 3 days, then oral PDN 60 mg for 7 days with tapering doses. At the third visit: BCVA LE = 20/ 40; color depth affection.
Borrego- Sanz L et al. [**10**]	66/ F	When waking up after 40 days in ICU (6 weeks)	Severe form; ICU for severe respiratory failure for 40 days	BCVA RE = 0.9, BCVA LE = HM; OCT LE: marked optic nerve head pallor; large cupping, without hemorrhages or edema; narrowing of the arterioles, thickness of the peripapillary RNFL correlated with significant temporal defect of the VF; RAPD in the LE, normal RE fundus	LE: Optic neuropathy in a patient with COVID-19	
Francois J et al. [**11**]	50/ F	2 days	Severe form, hospitalized for a severe bilateral pneumonia; she was suspected because of recent close contact with a fatal Covid 19 case	2 days after presentation: RE: blurring of vision accompanied by ocular congestion; temporal pain when moving the eyeball on day 8. BCVA RE = HM; BCVA LE = 0 logMar, RAPD; RE-VF: central scotoma, impaired color and contrast vision; BM RE: central nongranulomatous retrodescemetic precipitates and a mild inflammation in the anterior chamber. RE fundus-marked papillary edema, 2 peripapillary hemorrhages, mild vitreous inflammation, retinal vessel narrowing in the inferior retina.	RE: Optic neuropathy associated with panuveitis in COVID-19 infection	Local and general corticosteroids; Day 30: AFG-mild papillary edema and retinal vasculitis. After 1 month and a half: BCVA RE = + 2 log MAR (HM); normal anterior pole, without signs of inflammation, Fundus of RE: severe papillary atrophy.
Clarke KM et al. [**12**]	55/M	-	Severe form, fever 39.7 C0; blood pressure = 166/ 90 mmHg; respiratory frequency 40 beats/ minute; O2 was administered through nasal cannula, but it was not possible to maintain the saturation above 92% and therefore he was intubated and mechanically ventilated, taken to the ICU. Comorbidities: ex. smoker; HT; hypercholesterolemia.	After the cessation of the sedation, the patient had a profound, bilateral loss of vision, more pronounced at LE. Examination at bed BCVA RE = CF, BCVA LE = 3/ 30, IOP both eyes = 10 mmHg; relative APD in the RE. Fundus RE: papillary edema, splinter hemorrhages, which indicates sectorial NAION (infero-temporal); Fundus LE: mild papillary edema, temporal pallor, hemorrhages in the inferior sector. OCT both eyes - shortly after diagnosis - bilateral papillary edema, flame hemorrhages in the RE.	Both eyes - AION related to pronation in a patient with COVID-19 related ARDS	Mechanically ventilated in ICU, in pronation, with vasopressor medication, renal dialysis, blood transfusions. After 5 weeks: RE-VF: severe narrowing with preservation of macular vision; LE-VF: inferonasal loss with preservation of peripheral vision. After 2 months: OCT – both eyes: RNFL thinning with disc pallor. VA did not improve; the patient was advised to register to low vision support.
Golabchi N [**13**]	52/ M	2 weeks	Moderate form with fever, dyspnea, dry cough	7 days after discharge due to Covid: the sudden drop in VA in RE. BCVA RE = HM, BCVA LE = 9/ 10, normal BM exam; RE – RAPD Fundus of RE: pale disc, without edema, suggestive of a previous AION in antecedents; Fundus of LE: hyperemic optic disc. VF-RE: generalized depression with deep central and nasal scotoma, VF-LE: normal. OCT RE: diffuse thinning of the RNFL without macular edema.	RE – AION-a rare manifestation after COVID-19	Kaletra, Tamiflu, Hydroxychloroquine, Meropenem, Vancomycin, Tavanex. After 1 week: the patient is discharged with a good general condition, normal laboratory tests, no ocular complaints. After 4 weeks, irreversible optic atrophy in the RE.
Benito-Pascual B [**14**]	60/ F	-	-	LE: eye pain, conjunctival congestion and blurred vision. BCVA RE = 20/ 20; BCVA LE = 20/ 200; RAPD in LE. BM LE: panuveitis in AC with 3+, posterior synechiae. Fundus LE: vitritis 1+, papillary edema, subretinal fluid with peripapillary choroidal folds. OCT LE: RNFL edema.	LE- Panuveitis and ON as a possible initial presentation of Covid 19	Oral administration of PDN starting with a dose of 60 mg/ day; topical steroid every hour and mydriatics x 3/ day. Hydroxychloroquine 400 mg x 2/ day on day 1, then 200 mg/ day for 6 days; Kaletra 400 mg/ 100 mg x 2/ day for 10 days. After 15 days of hospitalization, at discharge: normal VA, anterior pole and eye fundus, without inflammatory signs; BCVA RE = 20/ 20, BCVA LE = 20/ 40. Fundus RE: C/ D 0.3, Fundus LE = C/ D 0.7 with pale optic disc; LE: VF defect. OCT LE: severe optic atrophy with RNFL and CGL thinning; PCR negative.
Assavapongpaiboon B, Jariyakosol S [**15**]	35/ F	1 week	Mild form, dry cough	Blurred vision and pain when moving the left eyeball. BCVA RE = 20/ 32; BCVA LE = CF; LE relative APD; Fundus both eyes-bilateral optic disc edema (LE > RE) CT scan of the brain and orbit: swollen optic nerve sheath; MOG + antibodies in serum.	Blurred vision and pain when moving the left eyeball. BCVA RE = 20/ 32; BCVA LE = CF; LE relative APD; Fundus both eyes-bilateral optic disc edema (LE > RE) CT scan of the brain and orbit: swollen optic nerve sheath; MOG + antibodies in serum.	MTPN 1 g/ day iv for 5 days, then oral prednisolone with slow tapering and oral Favipiravir for 5 days. 8 days after treatment: BCVA both eyes = 20/ 30; at 4 weeks: BCVA RE = 20/ 25; BCVA LE = 20/ 20 with a slight subjective residual dyschromatopsia in LE.
Sainath D [**16**]	56/ F	2 weeks	Mild form, fever, dry cough, isolation at home, treatment with vitamins	Both eyes- extraocular muscle movements – full but painful in superior and lateral gaze; BCVA = CF with alteration of the chromatic sense; BM and fundus of both eyes: normal; MRI of the brain and orbit: swelling of the right retrobulbar intraorbital segment of the optic nerve.	Acute bilateral retrobulbar ON - an atypical sequela of COVID-19	MTPN iv 250 mg x 4/ day for 3 days then oral MTPN 1 mg/ kg for 11 days according to ONTT After 7 days: BCVA both eyes = 6/ 9 (Snellen); VF both eyes: paracentral scotoma (30-2 Humpfrey) OCT LE RNFL normal thickness: 111 µm RE; 114 µm LE.
Sanoria A [**17**]	45/ M	4 weeks	Mild form, Comorbidities: well controlled DM II, HT	BCVA RE = 6/ 6; BCVA LE = 6/ 24; RAPD + to LE; Fundus RE: hyperemic papilla with blurred margins; Fundus of LE: pale, edematous papilla; OCT: increased RNFL (LE > RE); color vision and contrast sensitivity was reduced in LE. HumphreyVF central 30-2: inferior RE defect, inferior and superior LE defect; VEP: delayed latency of the P100 wave (120 ms RE, 225 ms LE) with reduced amplitude (6 µV RE, 1.7 µV LE); MRI brain and orbit: normal.	Bilateral sequential NAION post COVID-19	MTPN 1 mg/ kg for 6 days, then gradual, weekly dose reduction for 6 weeks. After one month of treatment: VF both eyes: persistent deficits; Fundus both eyes: disc pallor with gradual remission of edema.
Mahfuzullah MA [**18**]	45/ M	3 weeks	Mild form	BCVA RE = 3/ 60; BCVA LE = 6/ 6; RAPD in RE with impaired color vision; Fundus RE: papillary edema; Fundus LE – normal.	RE-ON post COVID-19	MTPN 1 g/ day for 3 days, then oral prednisolone; after treatment: BCVA RE = 6/ 9.
Mahfuzullah MA [**18**]	28/ F	4 weeks	Mild form	BCVA RE = 6/ 6; BCVA LE = 6/ 36; relative APD In LE with impaired color vision; Fundus LE: papillary edema in the nasal sector; Fundus RE - normal.	RE-ON post COVID-19	MTPN 1 g/ day for 3 days, then oral prednisolone; after treatment: BCVA RE = 6/ 9.
Mahfuzullah MA [**18**]	40/ F	8 weeks	Mild form, normal O2 saturation, fever, loss of taste and smell, without PCR	RE - blurring of vision for 15 days but without pain and congestion; BCVA RE = 3/ 6; relative APD in RE; impaired color vision; BCVA LE = 6/ 6; Fundus RE: papillary edema with star shaped macular exudate; fundus LE: normal.	RE-ON post COVID-19	MTPN 1 g/ day for 3 days, then oral prednisolone; after treatment: BCVA RE = 6/ 24.
*BCVA RE = best correction visual acuity right eye, BCVA LE = best correction visual acuity left eye, LP = light perception, RAPD = relative afferent pupillary defect, BM = biomicroscopy; IOP = intraocular pressure, AFG = angiofluorography, AAION = arteritic anterior ischemic optic neuropathy, PDN = prednisone, MTX = methotrexate, MTPN = methylprednisolone, RNFL = retinal nerve fiber layer, OCT = optical coherence tomography, LP = light perception, HM = hand motion, NSAID = non-steroidal anti-inflammatory drugs, CGL = cells ganglion layer, MRI = magnetic resonance imaging, CSF = cerebrospinal fluid, AQP4 = anti aquaporin4, MOG = myelin oligodendrocyte glycoprotein, AION = anterior ischemic optic neuropathy, VEP = visual evoked potential, CF = counting fingers, DM = Diabetes mellitus, HT = Hypertension, ON = Optic neuritis, VF = visual field, ICU = intensive care unit, AC = anterior chamber, MS = multiple sclerosis*						

*Pathophysiology*

Although the infection with Covid-19 virus mainly affects the respiratory system, ocular manifestations have also been described, from conjunctivitis with changes in the ocular surface (most of the cases) to diseases of the uvea, retina, optic nerve and neuro-ophthalmological complications [**19**,**20**]. The tropism of the Corona virus on the cranial nerves manifests itself on the sensory function, while the motor function is rarely affected [**21**]. In Sars-Cov-2 infection, complications generally occur within the second week after the onset of the symptoms [**13**]. There are two main mechanisms: a severe inflammatory response and a state of hypercoagulability, by activating the coagulation cascade [**22**]. In the first mechanism, pro-inflammatory factors such as C-reactive Protein (CRP), ferritin, cytokines (CK) and interleukins (IL) 2,6,7,10, and tumor necrosis factor (TNFɑ) are activated [**13**,**22**]. Regarding the neuro-ophthalmological manifestations associated with Covid-19, the physiopathogenic mechanisms involved are related to hypoxia, severe HT, toxic metabolic processes, ischemic stroke, hemorrhages associated with certain parainfectious and postinfectious inflammatory processes [**23**].

There are 3 theories: 

1. Postviral inflammatory syndrome - the sequel of a proinflammatory state with hypercoagulability and “CK storm”.

2. The result of certain systemic anomalies that include hypoxia and severe HT.

3. Direct viral invasion that seems to be less involved [**23**].

The coronavirus has a neurotropic and neuroinvasive character. The central nervous system (CNS) is reached in several ways: hematogenous/ lymphatic, in which the permanently infected leukocytes serve as a reservoir and at the same time as a vector in the propagation of the infection at the level of the CNS [**24**]. The way of dissemination is transneuronal, retrograde and follows the nasal infection and the involvement of the olfactory bulb [**25**]. The infection of the host cell is mediated by the angiotensin-converting enzyme 2 (ACE2) receptor of Sars-cov1 and Sars-cov2 [**26**,**27**].

The coronavirus shows a high tropism for ACE2 receptors that are present in endothelial cells and in most organs. Systemic endothelial dysfunction will induce a procoagulant state and ischemia with the triggering of venous/ arterial thromboembolic complications. In severe and moderate forms of the disease, the probability of these complications is higher, and their incidence is over 30% in the patients with Covid-19 infection [**28**]. So, thromboembolic events are the consequence of an excessive inflammatory phenomenon, endothelial dysfunction, platelet activation and stasis. This state of hypercoagulability with the formation of thrombi will lead to the compromise of the vascular circulation at the level of the eyeball, with the installation of ischemic phenomena in the form of ischemic optic neuropathies or central retinal vein occlusion (CRVO). The presence of ACE2 receptors at the level of neurons and vascular endothelium justifies the tropism of the Sars-Cov-2 virus for neuroepithelium and endothelium [**29**-**32**]. The virus was also detected in the nerves and neurons of autopsied patients, associated thrombotic episodes being also described [**30**-**32**]. At the ocular level, ACE 2 receptors are present in retinal cells: Muller cells, ganglion cells, photoreceptors, retinal vascular endothelium cells and choroidal cells [**33**].

*The association between ON and myelin oligodendrocyte glycoprotein (MOG) + antibodies* in the patients diagnosed with Covid-19 infection is still unclear. MOG is part of the class of glycoproteins, being located in the outermost area of myelin at the level of oligodendrocytes in the CNS. When MOG antibodies enter the CNS, MOG antibody disease (MOG-AD) is triggered. This disease is part of a group of demyelinating diseases, sometimes manifested only by the appearance of ON. MOG-AD was cited even before the emergence of the Covid-19 pandemic, being defined as a non-specific viral infection that includes prodromal symptoms in up to 61% of the patients [**34**]. Therefore, an immune-type reaction takes place, thus triggering an immune response associated with post-infectious demyelinating processes after various types of infections, such as *Herpes Simplex, Borellia, Epstein Barr*. The titer of MOG serum antibodies will increase because of the present infectious and inflammatory process, thus triggering the MOG disease through BBB rupture. The disease evolves unpredictably, monophasically or with periods of relapses, often mild ones, with a favorable prognosis compared to other demyelinating diseases [**6**]. The data from literature show that the titer of MOG antibodies is higher in the relapsed forms, compared to the remitted ones [**35**,**36**]; therefore, several hypotheses were issued: 1. Is ON caused by a postinfection? 2. Can the Covid-19 infection trigger or predispose to ON? Two hypotheses regarding the triggering of the first ON attack with MOG + antibodies are cited: 1. Covid antigen determines the formation of antibodies that will attack the protein in the external sheath of myelin, through a mechanism of molecular mimicry. A primary or secondary immune response will be triggered with variable duration, from a few hours even up to 10 days. The vast majority of ON cases started approximately one week after the diagnosis of Covid-19 infection, which supports this hypothesis. 2. The second hypothesis assumes a severe inflammation, accentuated by the growth of CK and the destruction of BBB, which determines the invasion of circulating anti-MOG antibodies. Thus, the sudden onset of ON after Covid-19 infection can be explained. The role of MOG antibodies is still unclear; they contribute to the regulation of the microtubule stability at the level of oligodendrocytes, acting as a cellular adhesive molecule [**37**].

*Optic neuropathies related to pronation*. In patients with Covid 19 infection and ARDS, prone positioning is a risk factor for the onset of ischemic optic neuropathy. Indeed, the prone positioning saves the life of the critical patient, but there is the risk of irreversible vision loss, which is minimized by the medical staff. Pronation favors intraocular pressure (IOP) fluctuations with hypoperfusion of the optic nerve. It produces an external compression of the orbit through a process similar to orbital compartment syndrome. We could also consider another indirect mechanism, increasing central venous pressure (CVP), with a consequence of increasing IOP due to the lack of valves at the level of the orbital veins [**38**]. The eye becomes very vulnerable to changes in position, with an increase in IOP along with an increase in CVP [**39**]. Other risk factors involved in the installation of ischemic phenomena of the optic nerve could be the medication initiated in the treatment of multiple systems organ failure (MSOF): sedatives, vasopressors, anesthetics, which alter the self-regulating mechanisms of optic nerve perfusion [**40**]. NAION can be prevented by positioning the patient in the inverted Trendelenburg position for 10 degrees, which can reduce IOP compared to the neutral position [**40**]. Immobilizing the patient on one side or malpositioning him causes pressure directly on the abdomen, with obstruction of the venous return to the heart. Trying to position the head at the same level with the heart or above the heart, helps to maintain the blood pressure within a range of 20% compared to the initial value and thus decreases the risk of installing AION [**41**].

## Results

In the articles in literature, an accurate diagnosis of postcovid optic neuropathy was carried out through an ophthalmological examination, in some cases associated with a neurological examination [**7**-**9**]. Imaging studies such as optical coherence tomography (OCT) [**5**,**7**,**9**,**10**,**12**,**13**,**15**,**17**], computer tomography (CT) [**15**], angiofluorography (AFG) [**2**,**11**], magnetic resonance imaging (MRI) [**3**-**5**,**7**-**10**,**16**-**18**], ultrasonography of the retrobulbar arteries [**2**], as well as laboratory, immunological and serological analyses were used. In order to clarify the etiological diagnosis and establish differential diagnoses with other infectious or autoimmune diseases, the following inflammatory tests were performed: erythrocyte sedimentation rate (ESR), ferritin, LDH, fibrinogen, CRP [**2**,**4**,**6**,**7**,**8**,**13**], D-dimers [**2**,**6**,**12**,**14**,**16**,**17**] immunological tests: rheumatoid factor (RF) [**2**,**4**,**6**,**7**,**8**,**11**,**13**,**14**,**15**], antinuclear antibodies (ANA), antineutrophil cytoplasmic antibodies (ANCA) [**6**,**7**], anti-aquaporin 4 antibodies (AQP4) [**6**], anti MOG IgG, antibodies IgG and IgM SARS-CoV-2; anti neuromyelitis optica antibodies (NMO), serology for *syphilis, tuberculosis, cytomegalovirus*, Lyme disease, *Ebstein Barr virus, Herpes Simplex*, human immunodeficiency virus, *Borellia*, hepatitis B and C virus, Mantoux skin test [**16**], QuantiFeron test for *tuberculosis* [**6**], vitamin B12 dosage [**4**], examination CSF with determination of oligoclonal bands [**3**,**5**,**7**].

21 patients affected by ON in the Covid-19 disease, 11 women and 10 men, were mentioned in the cited publications. The associated comorbidities were HT associated with DM [**2**,**8**,**17**], balanced, well controlled [**2**,**17**] or imbalanced [**8**] DM. Only one new case was diagnosed with DM [**6**]. Kirsty M et al. [**12**] described a severe case of post-Covid AION in a former smoker with HT and hypercholesterolemia.

From a clinical point of view, the optic neuropathies described by the authors were: retrobulbar (posterior) optic neuropathy in more cases [**7**,**16**,**18**], only one associated with MOG+ [**7**], anterior optic neuropathy (papillitis) [**6**,**7**], AION [**2**,**13**,**14**,**18**], from which one AAION [2] and the others NAION [**13**,**14**,**18**], one being related to pronation in an OTI patient with ARDS [**14**], neuroretinitis [**18**], suggested by the eye fundus with papillary edema with star-shaped associated with star-shaped macular exudates. It is difficult to specify the triggering mechanism of inflammatory optic neuropathies or ischemic optic neuropathies. Most of the time, the retrobulbar inflammatory damage had a favorable evolution under early intravenous (i.v.) corticosteroid treatment, with the recovery of visual acuity (VA) and perimetric deficits [**3**,**4**,**6**,**7**,**15**,**16**]. In the case of papillitis, the evolution was good according to the ON treatment trial (ONTT) scheme [**6**] or with preservation of VA, but OCT changes at the level of the retinal nerve fiber layer (RNFL) [**7**]. Despite the corticotherapy treatment, in many cases, the evolution towards optic atrophy was inevitable [**2**,**5**,**10**-**14**], with irreversible vision loss. 

Regarding the form of the Covid disease, most of the neuropathies described by the authors were found in the mild forms of the disease [**2**-**5**,**7**,**15**-**18**], but there were also some moderate forms [**6**,**8**,**13**] requiring oxygen and general treatment with antibiotics and anticoagulants. In several other cases, the Covid disease took on serious forms, with severe complications [**9**-**12**] and hospitalization in the intensive care unit (ICU) and even OTI [**12**]. 

The ocular symptomatology with the onset of physiopathogenic phenomena that heralds an ON started most of the time two weeks after the positivity of the nasopharyngeal PCR test [**4**,**5**,**7**,**9**,**13**,**16**], but there were also cases with the onset of the disease in two and a half weeks [**2**], in three weeks [**8**,**18**], four weeks [**6**,**17**,**18**], six weeks [**7**,**10**], two months [**18**], or later, after a period of six months [**7**]. There was a rarity of the onset of the ON in the first days after the positivity of the PCR test, respectively in two days [**11**] or in seven days [**3**,**15**]. In some cases, due to the serious condition of the patients, the authors could not specify the time interval from the positivity of PCR to the appearance of ON [**14**].

Regarding the age distribution of post-Covid ON in the studied patients, most were aged within 31-60 years: 31-40 years [**7**,**9**,**15**,**18**], 41-50 years [**4**,**8**,**11**,**17**,**18**], 51-60 years [**5**,**12**-**14**,**16**]. Few cases of ON were aged between 16 and 30 years [**3**,**7**,**18**] or 61-70 years [**2**,**6**,**10**]. A preponderance of ON post Covid was observed in the left eye (LE) [**2**,**3**,**5**,**7**-**10**,**14**,**18**], but without an important correlation between recovery and treatment; the right eye (RE) involvement was less common [**6**,**13**,**11**,**18**] and so was bilateral ocular involvement [**4**,**12**,**15**-**17**].

## Discussion

Although the Sars-Cov 2 virus mainly affects the respiratory system and can cause complications that are sometimes incompatible with survival, the neuro-ophthalmological complications should not be overlooked either. All the patients infected with Covid-19 must be monitored continuously and accurately, even the cases that were initially thought to be asymptomatic. Routine screening is mandatory to detect possible long-term neuro-ophthalmological implications. Serological, imaging and immunological tests are necessary to exclude other etiologies of ON. 

In patients with severe forms of Covid 19, who are in a prolonged pronation position in ICU sections, the risk of vision loss increases due to ischemic phenomena at the level of the optic nerve. Once CVP increases, as it is vulnerable to the changes in the patient’s position, the pressure of the orbital veins lacking valves with marked periorbital edema, also increase [**12**].

Hypoxia and hypercoagulability are risk factors in NAION. Since it is about a microvascular inflammation and a microembolic disease in NAION, anticoagulant therapy should be used carefully to the benefit of the patients with covid who are ventilated and are in pronation [**42**-**44**]. 

In multiple sclerosis (MS), Covid 19 is rather a precipitating factor or a triggering factor than a direct consequence of the infection itself [**8**]. The triggering or exacerbation of autoimmune diseases in patients with Covid 19 is continuously increasing in the specialty literature and further studies will guarantee the associative cause between MS and covid 19. In adults, ON is generally unilateral with good evolution in retrobulbar forms diagnosed early and treated with general corticosteroid therapy according to the ONTT scheme. In AION, the evolution is towards optic atrophy, with irreversible vision loss [**2**,**13**,**14**,**18**].

The specialty literature shows the results of several studies conducted among patients infected with Covid 19 during the pandemic: Jaafar et al. performed a quantitative meta-analysis of published studies on post-Covid neurological complications. Out of the 60 cases with Covid 19, of whom 40 (66.7%) were male and 18 (30%) were female, with an average age of 44.95 years, ON was found in 7 patients out of whom 4 were men. The forms of Covid were mild for four patients, who were not hospitalized, and three patients were hospitalized [**45**]. In older adults, isolated inflammation of the optic nerve is rare encountered and generally takes the form of a papillitis; in elderly people, ON is generally associated with a systemic disease or another granulomatous disease rather than with a previous viral infection [**46**].

Doria et al. [**23**] studied the complications of coronavirus infection in 2019. In a study carried out in a hospital in Spain, they described a single case of ON, which started during the recovery phase of the disease [**47**]. Cases with MOG+ antibodies were also reported both in patients with suspected covid [**48**] and in those with confirmed covid disease [**49**]. 

Buravej et al. [**15**] reported 9 cases of ON post Covid with MOG+ antibodies; only one case with relapse, the other 8 being at the first set. Zhou et al. [**49**] described a case of bilateral ON associated with peripheral retinal hemorrhages with good therapeutic response to iv corticosteroid therapy. A case of acute disseminated encephalomyelitis with ON and other neurological changes was also described [**50**], and Sardar et al. [**51**] described an ON associated with idiopathic ICH in a patient with Covid infection. 

In patients already diagnosed with MS, a possible aggravation of the disease was observed, as well as an increased probability for a possible relapse of an ON attack. The specialty literature also published a case of MS that followed Sars-Cov 2 infection in a patient with inflammation of the right optic nerve and demyelinating lesions at the level of the CNS [**52**]. 

## Conclusions

The number of publications related to ophthalmological pathology in general and neuro-ophthalmological changes in particular, in the context of the infection with Covid-19 is limited, reduced in number, compared to other complications. These were discovered either during the active period of the disease or during the recovery period. The rarity of cases could be explained by the lack of safety against the infection with Covid-19 and the difficulty of performing an ophthalmological examination at the bedside or in ICU.

The ocular manifestations of the Covid infection can be diverse in terms of symptomatology and prognosis, therefore, a complete ophthalmological examination is required for any Covid + patient who has symptoms in the ophthalmological sphere.

**Conflict of interest statement**

The authors state no conflict of interest.

**Acknowledgements**

None.

**Sources of Funding.**

None.

**Disclosures**

None.
